# Pulmonary Tuberculosis and Drug Resistance in Dhaka Central Jail, the Largest Prison in Bangladesh

**DOI:** 10.1371/journal.pone.0010759

**Published:** 2010-05-21

**Authors:** Sayera Banu, Arman Hossain, Mohammad Khaja Mafij Uddin, Muhammad Reaj Uddin, Tahmeed Ahmed, Razia Khatun, Asif Mujtaba Mahmud, Khurshid Alam Hyder, Afzalunnessa Binte Lutfor, Md. Sirajul Karim, Khalequ Zaman, Md. Ashraful Islam Khan, Pravat Chandra Barua, Stephen P. Luby

**Affiliations:** 1 International Centre for Diarrhoeal Disease Research, Bangladesh, Dhaka, Bangladesh; 2 National Institute of Diseases of the Chest & Hospital, Mohakhali, Dhaka, Bangladesh; 3 World Health Organization (WHO) Bangladesh, Mohakhali, Dhaka, Bangladesh; 4 Department of Microbiology, Sir Salimullah Medical College, Dhaka, Bangladesh; 5 ADMS, Savar Cantonment, Savar, Dhaka, Bangladesh; 6 Directorate of Prisons, Dhaka, Bangladesh; 7 National TB Control Programme, Directorate General of Health Services, Mohakhali, Dhaka, Bangladesh; MRC Laboratories, Gambia

## Abstract

**Background:**

There are limited data on TB among prison inmates in Bangladesh. The aim of the study was to determine the prevalence of pulmonary tuberculosis (TB), its drug resistance and risk factors in Dhaka Central Jail, the largest prison in Bangladesh.

**Methods:**

Cross sectional survey with, active screening of a total number of 11,001 inmates over a period of 2 years. Sputum samples from TB suspects were taken for acid- fast bacilli (AFB) microscopy, culture and drug susceptibility testing.

**Results:**

Among 1,781 TB suspects 245 (13.8%) were positive for AFB on microscopy and/or culture. The prevalence rate of sputum- positive pulmonary TB was 2,227/100,000. Fifty three cases (21.6% of 245 cases) were AFB- negative on microscopy but were found positive on culture. Resistance to isoniazid, rifampicin, streptomycin and ethambutol was 11.4%, 0.8%, 22.4% and 6.5% respectively. No multidrug resistance was observed. The main risk factors of TB in prison were exposure to TB patients (adjusted odds ratio 3.16, 95% CI 2.36–4.21), previous imprisonment (1.86, 1.38–2.50), longer duration of stay in prison (17.5 months for TB cases; 1.004, 1.001–1.006) and low body mass index which is less than 18.5 kg/m^2^ (5.37, 4.02–7.16).

**Conclusions:**

The study results revealed a very high prevalence of TB in the prison population in Dhaka Central Jail. Entry examinations and active symptom screening among inmates are important to control TB transmission inside the prison. Identifying undiagnosed smear-negative TB cases remains a challenge to combat this deadly disease in this difficult setting.

## Introduction

Tuberculosis (TB) has been a major health problem in penitentiary systems all over the world. The extent of TB in prisons, which is much greater than in the community, is often underreported. Prevalence of TB in prison is often five to 10 times higher than the national rates [1], [2], [3] in some cases, by as much as 30 to 50-folds [4], [5]. The high number of imprisoned persons, extremely overcrowded conditions, inadequate ventilation, and poor general health of inmates facilitate the spread of tuberculosis. Prisons represent a high risk setting for development and transmission of multidrug-resistant (MDR) TB [6]. In many countries where the incidence of tuberculosis is very high, prisons have been found to play a significant role in the epidemiology of drug resistant tuberculosis [1], [7], [8]. Data on the prevalence of TB in prisons in Bangladesh are limited. The Bangladesh National Tuberculosis Control Programme (NTP) has taken initiatives to implement directly observed treatment – short course (DOTS) in some prisons of Bangladesh in 2002. These are the cases that come to the outpatient service of the prison hospital seeking treatment. Drug resistance patterns in these cases are not known as culture and sensitivity-testing of sputum samples are not performed in the prison setting.

The aim of this study was to determine the prevalence of TB and drug-resistant TB in the largest prison in Bangladesh, the Dhaka Central Jail, and to assess risk factors associated with the development of TB among the prisoners.

## Methods

The study was undertaken in the Dhaka Central Jail which is home to about 11,000 inmates at any time although it has a capacity for only 2,600 inmates. There was no entry point screening system for TB before the study. We screened 11,001 inmates over a period of 24 months from October 2005 to September 2007. A total of 11,000 inmates consented to participate in the study. Only one inmate refused to participate; he had access to the prison hospital that also provides diagnostic services and treatment of TB. A medical doctor interviewed the inmates (20–30 inmates/day), examined all TB suspects (having cough for more than three weeks) and collected information about risk factors for development of TB in the jail. The Dhaka Central Jail consists of 17 building blocks. We started screening in one block and successively completed screening in other blocks. During the study period, inmates of all the buildings were screened once. Fifteen buildings were visited another time for screening the inmates except those who had already been screened earlier. The inmates were interviewed at any point of their stay in prison. There are two distinct areas for male and female. Among the 11,000 inmates, 768 were female.

Three sputum samples, one spot and two early morning samples, were collected from all suspects and brought to International Centre for Diarrhoeal Disease Research, Bangladesh (ICDDR,B) Tuberculosis Laboratory for acid-fast bacilli (AFB) microscopy, culture and drug sensitivity. Diagnosis was based upon the demonstration of AFB in sputum samples subjected to Ziehl-Neelsen staining under light microscopy, or positive on culture using Löwenstein-Jensen solid media. Drug sensitivity testing was done using the proportion method [9]. The concentrations of anti-TB drugs tested were as follows: isoniazid 0.2 mg/l, rifampicin 40 mg/l, ethambutol 2 mg/l and streptomycin 4 mg/l. An isolate was considered as resistant to a particular antibiotic when the number of colonies on the drug-containing medium was 1% or more of the number developing on the drug-free medium. Diagnosed TB cases were isolated and treated by the Jail Hospital following DOTS guidelines.

The prevalence of TB in jail was defined as the number of cases diagnosed during the study period divided by the total number of inmates screened during that period multiplied by 100,000, and expressed as cases per 100,000.

To determine potential risk factors for development of pulmonary TB in jail, all screened inmates with no history of cough exceeding three weeks were taken as controls. Data collected for each inmate were: date of entry into the prison, date of interview and sample collection, age, height, weight, address, occupation, symptoms of TB, smoking, drug use, previous stay in prison, exposure to TB patient and previous history of TB disease. Data were entered into the computer using SPSS, version 11.5. Odds ratios (OR) and their 95% confidence intervals (CI), were estimated using binary logistic regression, with TB positivity as an outcome. Univariate analyses were performed to examine the effect of each variable on the risk of TB. Multivariate model was then constructed, including variables that showed an effect on the prediction of TB positivity in the univariate analyses at the p = 0.05 level of significance.

Inmates were asked about their occupation before entering the prison. Most of them were of low socioeconomic conditions. They were categorized as self-employed which included farmers, tailors, hawkers etc., service where a monthly salary is paid, and, shop owners and others engaged in the construction, garment, transport, and export-import sectors etc. were included as business.

The prisoners were asked about smoking and drug abuse. An inmate was considered as smoker who took five cigarettes per day regularly. Those who reported regular taking of *Cannabis or Ganja*, Phensidyl (a liquid cough remedy), heroin, alcohol, sleeping pills etc. at any period of their life were considered as substance abusers. Occasional users were not included.

Body mass index (BMI) was calculated as the individual's body weight in kg divided by the square of their height in metre. Height and weight of every screened inmate were taken during the interview by using portable stadiometer and bathroom weight scale respectively. Nutritional status was classified as severe malnutrition (BMI<16.0), moderate malnutrition (BMI = 16.0–16.9), marginal malnutrition (BMI = 17.0–18.5), normal (BMI = 18.5–24.9), and overweight (25 or more).

The study protocol was approved by the Ethics Review Committee of ICDDR,B. All study subjects provided written informed consent. The study staff explained to the inmates about TB, the need for screening, the benefits of receiving treatment if TB is detected, and the voluntary nature of participation in the study. All questions of the inmates were answered to their satisfaction.

## Results

The study team screened 11,000 inmates (10,232 male and 768 female) over a period of 24 months; 1,781 inmates (1,685 male and 96 female) with cough of at least 3 weeks duration were suspected to have TB. Among 1,781 suspects 192 (10.8%) cases were positive by AFB on microscopy and 245 cases were positive by culture. Fifty-three AFB-negative cases were positive by culture. Only 4 confirmed cases were female. The prevalence of sputum positive (AFB and/or culture) TB was 2,227/100,000.

Sixty-nine (n = 170) percent of all strains were susceptible to all first line antitubercular drugs and resistance to any drug was observed in 30.6% (n = 75) of strains (Table 1). Eleven percent of the strains were resistant to INH, 0.8% were rifampicin-resistant, 22.4% were streptomycin and 6.5% were ethambutol-resistant. No MDR TB was found in the prison population.

**Table 1:** Anti tuberculosis drug resistance pattern of *Mycobacterium tuberculosis* isolates.

Drug	Total cases n = 245	Percentage (%)
Streptomycin	55	22.4
Isoniazid	28	11.4
Rifampicin	02	0.8
Ethambutol	16	6.5
Any drug	75	30.6
Any one drug	51	20.8
Any two drugs	22	9.0
Any three drugs	2	0.8

Most of the inmates, both TB (75.9%) and non-TB (80.2%) subjects, were from urban areas (Table 2). Forty three percent were categorized as self-employed and 43.6% were engaged in business or service before imprisonment. Fifty-six percent of the prisoners were between 21–30 yrs of age.

**Table 2:** Socio demographic characteristics of TB and non-TB cases and odds ratios (95% CI) for risk factors for tuberculosis.

Characteristics	Subcategory	Inmates with tuberculosis, n (%)	Inmates without tuberculosis, n (%)	OR (95% CI)	AOR (95% CI)
Sex	Male	241 (98.4)	9991 (92.9)	1.0	
	Female	04 (1.6)	764 (7.1)	0.22 (0.081–0.587)	
Place of residence	Rural	59 (24.1)	2134 (19.8)		
	Urban	186 (75.9)	8618 (80.2)		
Age	10–20 y	16 (6.5)	1463 (13.6)	1.0	
	21–30 y	137 (55.9)	5833 (54.3)	2.15 (1.26–3.61)	
	31–40 s	63 (25.7)	2330 (21.7)	2.47 (1.42–4.29)	
	more than 40 y	29 (11.8)	1124 (10.5)	2.35 (1.27–4.36)	
Occupation	Self-employed	110 (44.9)	4599 (42.8)		
	Business	53 (21.6)	2220 (20.6)		
	Service	54 (22.0)	2296 (21.4)		
	Unemployed	25 (10.2)	1008 (9.4)		
	Housewife	3 (1.2)	630 (5.9)		
Smoking	No	35 (14.3)	2795 (28.6)	1.0	1.0
	Yes	210 (85.7)	6981 (71.4)	2.40 (1.68–3.45)	1.42 (0.96–2.10)
History of drug abuse	No	162 (66.1)	6222 (67.6)		
	Yes	83 (33.9)	2985 (32.4)		
Previously diagnosed as TB	No	185 (75.4)	9416 (96.4)	1.0	1.0
	Yes	60 (24.5)	352 (3.6)	8.68 (6.36–11.83)	3.25 (2.28–4.64)
Reported weight loss (in jail)	No	53 (21.6)	8903 (91.1)	1.0	
	Yes	192 (78.4)	870 (8.9)	37.07 (27.14–50.65)	
Previous stay in prison	No	158 (64.5)	7985 (81.7)	1.0	1.0
	Yes	87 (35.5)	1783 (18.3)	2.47 (1.89–3.22)	1.86 (1.38–2.50)
Exposure to TB patient	No	111 (46.1)	7972 (81.7)	1.0	1.0
	Yes	130 (53.9)	1789 (18.3)	5.22 (4.03–6.76)	3.16 (2.36–4.21)
Duration of stay in prison (month)	Median (Range)	17.50 (0.03–200.7)	5.13 (0.03–314.9)	1.006 (1.003–1.009)	1.004 (1.001–1.006)
BMI	Normal (18.5–24.9)	63 (26.0)	6576 (61.3)	1.00	1.0
	Overweight (≥25)	01 (0.4)	495 (4.6)	0.21 (0.30–1.52)	0.25 (0.34–1.80)
	Marginal (17.0–18.5)	50 (20.7)	2304 (21.5)	2.27 (1.56–3.29)	2.03 (1.38–2.98)
	Moderate (16.0–16.9)	57 (23.6)	866 (8.1)	6.87 (4.77–9.90)	5.15 (3.50–7.55)
	Severe (<16.0)	71 (29.3)	492 (4.6)	15.06(10.60–21.41)	10.96 (7.54–15.95)

OR = odds ratio; AOR = adjusted odds ratio; CI = confidence interval.

A number of risk factors were associated with development of TB among the prisoners (Table 2). Those who had a history of TB previously had three-fold higher risk than those who did not. Risk was also three times higher among those with a history of exposure inside as well as outside the prison. Of 130 inmates 97 (75%) had a history of contact in prison, 26 (20%) in family and 7 (5%) in the community. Previous stay in prison was also associated with increased risk (1.8 times) of developing TB. Risk of having TB was slightly higher among those who had smoked. Among the TB cases, 85.7% had the history of taking at least 5 cigarettes per day regularly compared to 71.4% among non-TB cases. However, there was no risk observed among those who had used illicit drugs. A substantial number of inmates were also found to be intravenous drug users. Malnutrition, as defined by low BMI (<18.5), was also associated with TB; this can either be a risk factor or result of TB. Moderately malnourished inmates were five times more likely to have TB. The likelihood of TB was 11 times more in inmates with severe malnutrition (Table 2). Fifty-three percent of all TB cases had BMI<17. Overweight inmates were less likely to have TB (OR 0.25, 95% CI 0.34–1.80). The median duration of stay in prison (17.5 months) was much higher for the inmates diagnosed as having TB than that of non-TB subjects which is around 5 months (p = 0.001). Longer duration of stay in prison was associated with increased risk of developing TB among the prisoners (Table 2).

Among the 245 TB cases identified during the 24 months period, 37% were detected within six months of imprisonment suggesting that a substantial number of inmates might enter the prison with active TB or in the early stage of the disease or develop TB within six months. Among them about 50% had a history of previous imprisonment.

## Discussion

Our study has identified a substantial number of TB cases in Dhaka Central Jail over the 24 months period. The prevalence rate was 2,227 per 100,000 which is over 20-fold higher than the rate in the general population [10]. There is limited data on the prevalence of TB from urban areas. However, in a recently done survey, the prevalence rate of smear-positive TB in urban areas was 49.9 per 100,000 (unpublished data). TB rates in prison in some Asian countries were several times higher than in the general population [1], [11], [12], [13]. In Thailand the rate was 568/100,000 and in Pakistan it was 657/100,000 which are eight and 3.75 times higher than the national rate, respectively. However, the rate found in this study is extremely high, which needs to be addressed immediately, and a more aggressive approach to detecting and controlling TB in prisons is necessary.

A considerable proportion (21%) of culture-positive cases were AFB microscopy-negative. Studies show that culture increased the rate of detection of mycobacteria in smear negative pulmonary TB by 25 to 38% [14], [15], [16], [17]. Under the DOTS program only AFB microscopy is being done. This study identified substantial number of smear-negative culture positive tuberculosis cases, which demonstrated that sputum culture was useful in this setting. Facilities like culture and chest X-ray (CXR), which can be helpful in detecting suspected smear-negative TB cases, are not available in the prison system in Bangladesh. Most countries rely on CXR followed by sputum analysis for suspects in prisons which is more efficient than symptom screening alone [16]. We might have not been able to detect some of the asymptomatic TB cases who did not have cough for three weeks.

Drug resistance was not common in the prison; no MDR case was identified. In many countries, where TB has been a major heath problem in prison settings, multidrug resistance was found to be high among both new and previously treated cases [7], [8], [18], [19]. The drug susceptibility results from our study will help the NTP to formulate and implement the TB control policy for prisons. This will improve the treatment of the TB cases in jail also and ensure completion of treatment after release from jail. This would prevent emergence of drug resistance and worsening of the situation, as has occurred in prisons in many Russian, and other countries [18], [19], [20], [21].

Our study measured the risks associated with tuberculosis in the prison population. The disease risk was increased for inmates who had a previous history of suffering from TB. The average time between the onset of recent disease, and the completion of previous treatment was around 48 months indicating that the increase in the number of TB cases might be largely due to reactivation and reinfection. Our data also showed that a considerable number (37%) of TB cases were identified during the first six months of their stay in prison confirming that independent of incarceration, prison inmates run a higher risk of developing active TB than the general population, which might be one of the main reasons for the high incidence of tuberculosis observed in prisons. Again the disease risk was greater for persons who stayed in the prison for longer periods and with a history of previous imprisonment. According to our data the risk of infection was more than three times higher among those who were living with an inmate with TB strongly suggesting that transmission inside the prison also contributes to the high prevalence.

Research has shown an association between malnutrition and TB [22], [23], [24] and in our study we confirmed this in the prison population in Bangladesh. The likelihood for having TB was lower for overweight people compared to inmates with normal weight. The very design of the study does not allow us to conclude whether TB results in malnutrition or malnutrition predisposes to TB. But the presence of malnutrition in such a population is an important screening indicator of the disease in this population. Unlike what was found in prisons in Russia [25], the use of illicit drugs was not a risk factor associated with TB in Dhaka Central Jail.

The results of this study reveal a very high prevalence of TB in the Dhaka Central Jail that needs to be addressed urgently. It is evident that the spread of the disease occurs within the prison and it is very likely that it would also spread to the community at large after prisoners are released. Screening for TB symptoms is highly recommended as the first line of defense in the prison. Considering that a large group of inmates enter the prison with the disease, srceening procedures should be in place at the time of admission into the prison. Prisons provide a unique opportunity to srceen and treat a population at high risk that might not otherwise have access to medical care. This could help control the disease effectively in the prison as well as in the community. However, there is one difficulty in securing follow-up of TB cases who are released before completion of their treatment. Although at the time of release they are given a card with the treatment details and are asked to report to their nearest DOTS centre, it remains unknown whether they continue the treatment or not.
